# Peptides from Cauliflower By-Products, Obtained by an Efficient, Ecosustainable, and Semi-Industrial Method, Exert Protective Effects on Endothelial Function

**DOI:** 10.1155/2019/1046504

**Published:** 2019-02-06

**Authors:** C. Caliceti, A. L. Capriotti, D. Calabria, F. Bonvicini, R. Zenezini Chiozzi, C. M. Montone, S. Piovesana, M. Zangheri, M. Mirasoli, P. Simoni, A. Laganà, A. Roda

**Affiliations:** ^1^Department of Chemistry “Giacomo Ciamician”, Alma Mater Studiorum-University of Bologna, Bologna, Italy; ^2^Department of Chemistry, Università di Roma “La Sapienza”, Rome, Italy; ^3^Department of Pharmacy and Biotechnology, Alma Mater Studiorum-University of Bologna, Bologna, Italy; ^4^Department of Medical and Surgical Sciences, Alma Mater Studiorum-University of Bologna, Bologna, Italy

## Abstract

The large amount of cauliflower industry waste represents an unexplored source of bioactive compounds. In this work, peptide hydrolysates from cauliflower leaves were characterized by combined bioanalytical approaches. Twelve peptide fractions were studied to evaluate unexplored biological activities by effect-based cellular bioassays. A potent inhibition of intracellular xanthine oxidase activity was observed in human vascular endothelial cells treated with one fraction, with an IC50 = 8.3 ± 0.6 *μ*g/ml. A different fraction significantly induced the antioxidant enzyme superoxide dismutase 1 and decreased the tumor necrosis factor *α*-induced VCAM-1 expression, thus leading to a significant improvement in the viability of human vascular endothelial cells. Shotgun peptidomics and bioinformatics were used to retrieve the most probable bioactive peptide sequences. Our study shows that peptides from cauliflower waste should be recycled for producing valuable products useful for the prevention of endothelial dysfunction linked to atherogenesis progression.

## 1. Introduction

Agricultural and food waste management is a great challenge for global security and environmental governance, directly linked with global competitiveness, increasing population and other economic related factors. Under the European 2020 growth strategy launched in 2010, Europe has set itself the goal of shifting from linear to circular models of production and consumption. This important issue needs advanced efficient alternatives other that landfilling or composting, in order to maximize the value derived from such an important waste source. The food waste, including both edible food and inedible parts, has been estimated in Europe of 88 million tons (9 million tons comes from primary production) directly associated with around 143 billion euros of costs [1].

In the last decade, an increasing attention has been devoted to the recycling of protein or other functional ingredients from fruit and vegetable by-products. In the perspective of biosustainable development and renewable resource technologies, by-products and waste represent a relatively cheap source of material suitable for bioactive molecules production [2], which would reduce both the amount of waste and the related costs of disposal, while producing value-added nutritional products [3]. Indeed, leaf protein has been considered as a supplementary protein source since the 1960s [4, 5]. In particular, food processing wastes and by-products have been considered for the production of antioxidant and ACE inhibitor peptides [3]. These peptides are often functionally inactive within the native proteins and must be released by proteolysis (*in vivo* digestion, *in vitro* enzymatic hydrolysis, or bacterial fermentation) to achieve their potential “bioactive” roles.

As a representative example, the cultivation and consumption of cauliflower *(Brassica oleracea L. ssp botrytis)* have increased rapidly over the last few years with a large waste production, except for cauliflower curd (the sole edible part of cauliflower). Tons of cauliflower by-products (stems and leaves) are also generated during the harvest every year. Cauliflower is well known to contain various beneficial molecules, such as vitamin C, glucosinolates, carotenoid, and leaf protein [6, 7]. Numerous extraction techniques have been developed for bioactive compound extraction, such as supercritical fluid extraction [8], microwave-assisted extraction [9], and ultrasonic-assisted extraction [7], in order to treat larger quantities at the industrial scale still controlling the cost of the entire process.

Protein hydrolysates from cauliflower by-products have shown antioxidant [10] and angiotensin I-converting enzyme (ACE) inhibitory [11] activities in cell-free systems; therefore, they may be potential complementary to antihypertensive drugs [12]. It has also been reported that they regulate the glucose consumption and glycogen content in HepG2 cells, indicating an important role also in glucose metabolism [7]. In addition, several authors studied numerous antimicrobial peptides from plants, such as thionins, defensins, proline-rich peptides, lipid transfer proteins, cyclotides, and snakins [13, 14] that are also found in *Brassicaceae* species [15].

However, few researchers have focused on the study of protein fractions and preparation of their hydrolysates from cauliflower by-products and its biological activities [7, 11, 16, 17] in order to exploit them as preventive biomolecules for people genetically predisposed to diseases or within the framework of a healthy lifestyle.

Therefore, the aim of this paper was the development of a combined “ad hoc” bioanalytical approach based on an efficient recovery of peptides from cauliflower leaves, a characterization of their functional properties as potential nutraceuticals with highly predictive effect-based bioassays in cells and an in silico identification of the most active peptides.

The study of peptide bioactivity, with highly predictive cell models, is an efficient and reliable tool to reproduce *in vivo* physiological conditions avoiding the use of animal experiments to observe their effects on a wide range of biological activities, from endothelial dysfunction to antimicrobial properties.

## 2. Materials and Methods

### 2.1. Materials/Chemicals

Xanthine oxidase from bovine milk, luminol sodium salt, xanthine, oxypurinol, PBS tabs, Na-EDTA salt, gelatin from bovine skin, penicillin/streptomycin, trypsin-EDTA, Trolox, and 2′,7′-dichlorodihydrofluorescein diacetate (H_2_DCFDA) were purchased from Sigma-Aldrich (St. Louis, MO, USA). Sodium perborate, boric acid, NaOH, and FeCl_2_ were from Carlo Erba (Milan, Italy). M200 medium, low serum growth supplements, and fetal bovine serum, RNaseOUT, were purchased from Thermo Fisher Scientific (Waltham, MA, USA). RNeasy Mini Kit was from QIAGEN (Hilden, Germany). Primers for RT-PCR were purchased from IDT (Coralville, IA, USA). Cell counting kit-8 (CCK8) and LDH assay kit were purchased from Dojindo Molecular Technologies (Rockville, MD, USA). SuperScript® III First-Strand Synthesis SuperMix and EXPRESS SYBR® GreenER™ qPCR SuperMix were purchased from Life Technologies (Carlsbad, CA, USA). All the other chemicals and solvents were of the highest analytical grade.

### 2.2. Peptidomic Workflow

The entire peptidomic workflow was performed as previously reported [17] with some modifications. The procedure is reported in Supplementary Material S1. Briefly, 1 kg of lyophilized cauliflower by-products was extracted using an ecofriendly saline buffer consisting of 50 mmol L-1 Tris-HCl (pH 8.8) and 15 mmol L-1 KCl. The extracted proteins were digested by Alcalase® enzyme and the whole obtained hydrolysate was purified by a semipreparative reverse phase high-performance liquid chromatography (SP-RP-HPLC) in order to simplify the complex mixture. Twelve fractions were collected and subsequently tested for specific and less unexplored bioactivities. The fractions with positive bioactivity were further analyzed by nano-HPLC coupled to high-resolution mass spectrometry. The peptides in the most active fractions were identified by peptidomic technologies and screened for bioactivity by the use of bioinformatics, to retrieve most probable bioactive peptide candidates.

### 2.3. Sample Preparation for Analysis

Stock solutions were prepared solubilizing cauliflower lyophilized fractions derived from HPLC separation in 1 ml of PBS buffer 0.1 M pH 7.4 by sonication. The protein content for each stock solutions was determined by absorbance spectroscopy, at 280 nm by NanoDrop 2000c (Thermo Fisher Scientific Inc., Massachusetts, USA). Stock solutions were diluted in PBS buffer 0.1 M pH 7.4 to obtain a final concentration of 10 mg/ml in protein content. After filtration, fractions were sampled and stored at -20°C for further analysis.

### 2.4. Cell Culture

In order to study the protective effect of peptide fractions on endothelial dysfunction, experiments were performed in human umbilical vein endothelial cells (HUVECs), a robust *in vitro* model for the study of endothelial cell physiology and function [18].

HUVECs pools, purchased from Life Technologies, were plated on gelatin-coated tissue culture dishes and maintained in phenol red-free basal medium M200 (Life Technologies) containing 10% FBS and growth factors (LSGS, Life Technologies) at 37°C with 5% CO_2_. Cells from passages 3 to 7 were actively proliferating (70–90% confluent) when samples were harvested and analyzed [19].

### 2.5. Cell Viability Bioassay

The cell viability was assessed by WST8 [2-(2-methoxy-4-nitrophenyl)-3-(4-nitrophenyl)-5-(2,4-disulfophenyl)-2H-tetrazolium, monosodium salt] (Dojindo Molecular Technologies, Japan) that, in the presence of an electron mediator, is reduced by dehydrogenases in cells (as a vitality biomarker) to formazan dye which is soluble in the tissue culture medium. The amount of the formazan dye generated by dehydrogenases in cells is directly proportional to the number of living cells [20]. The decrease in absorbance between the treatment after 24 h (representing *t*_1_) and the control (representing *t*_0_) was monitored at 37°C at 450 nm using a Varioskan™ flash multimode reader.

HUVEC cells were seeded in a transparent 96-well plate at a density of 5 × 10 cells/well [3]. The next day, cells were treated with stock dilutions (1 × 10^0^-1 × 10^−2^ mg/ml in protein content) in complete culture medium for 24 h.

### 2.6. Cell Cytotoxicity: Lactate Dehydrogenase Release

Lactate dehydrogenase (LDH) release from HUVECs was monitored by collecting aliquots of medium at different times, using a standard spectrophotometric method [16]. The method is based on a coupled enzymatic reaction in which LDH catalyzes the conversion of lactate to pyruvate via NAD^+^ reduction to NADH. Diaphorase reduces tetrazolium salt, oxidizing NADH, to a red formazan product that can be measured at 490 nm. Medium derived from HUVECs treated with stock dilutions of protein fractions (1 × 10^0^-1 × 10^−2^ mg/ml in protein content) for 24 h was collected and the increase in absorbance between the treatment after 24 h (representing *t*_1_) and the control (representing *t*_0_) was monitored at 37°C using a Varioskan™ flash multimode reader.

### 2.7. Intracellular Total Oxidant Fluorescent Detection

Intracellular oxidant levels were evaluated by using the oxidant-sensitive fluorescent probe 2′,7′-dichlorodihydrofluorescein diacetate (H_2_DCFDA).

Briefly, the probe is not fluorescent until the acetate groups are removed by intracellular esterases; in the presence of oxidants, the probe is oxidized within the cells producing a fluorescent signal related to intracellular oxidant levels that was measured using a microtiter plate reader (Varioskan™ flash multimode reader, Thermo Fisher Scientific). Excitation wavelength was 485 nm and emission wavelength was 535 nm. HUVECs were treated with cauliflower peptide fractions (1 × 10^0^-1 × 10^−2^ mg/ml) for 24 h and Trolox (100 *μ*M) was used as reference. After treatment, cells were incubated with 5 *μ*M H_2_DCFDA for 20 min at 37°C and then subjected or not to oxidative stress generated by 50 *μ*M H_2_O_2_ for 30 min. The decrease of fluorescence signal between cells treated with cauliflower peptide fractions and control was reported as the percentage of intracellular reactive oxygen species (ROS) normalized with H_2_O_2_ treatment alone [21].

H_2_DCFDA can be used as a redox indicator probe for detecting intracellular oxidant formation caused by changes in iron or heme signaling or peroxynitrite (ONOO^−^) formation. The fluorescent response based on the oxidation of DCFH provides an index for the total oxidants present in biological systems, not for cell-derived H_2_O_2_. This limitation determines a low selectivity toward H_2_O_2_ [22–24].

### 2.8. RNA Extraction

HUVECs were preincubated with cauliflower peptide fractions (1 mg/ml) for 8 hours at 37°C before 24 h of exposure to TNF-*α* (10 ng/ml). Total RNA was extracted using a commercial RNA extraction kit (QIAGEN) [20].

### 2.9. Real-Time PCR

RNA concentration and purity were determined by NanoDrop 2000 spectrophotometer (Thermo Fisher Scientific, Waltham, MA). 25 ng of total RNA was reverse transcribed using the SuperScript® III First-Strand Synthesis SuperMix (Life Technologies, Carlsbad, CA, USA) and amplified using the EXPRESS SYBR® GreenER™ qPCR SuperMix (Life Technologies, Carlsbad, CA, USA) according to the manufacturer's protocol in a final volume of 20 *μ*l. Real-time PCR reactions were conducted on a Rotor-Gene Q QIAGEN Real-Time PCR System (QIAGEN GmbH, QIAGEN Strasse 1, D-40724, Hilden), with an initial 5 min incubation at 60°C, then 2 min at 95°C, followed by 40 cycles of amplification: 95°C for 15 s and 60°C for 1 min and examined on by Rotor-Gene Real-Time Analysis Software 6.0 (QIAGEN GmbH, QIAGEN Strasse 1, D-40724, Hilden). Primer concentration was 500 nM. The following primers were used: LOX1: forward 5′-TCGGGCTCATTTAACTGGGAA-3′, reverse 5′-TTGCTGGATGAAGTCCAGATCA-3′; NOX2: forward 5′-GTCTCAGGCCAATCACTTTGC-3′, reverse 5′-CATTATCCCAGTTGGGCCGT-3′; NOX-4: forward 5′-TCTGGCTCTCCATGAATGTCC-3′, reverse 5′-GACACAATCCTAGCCCCAACA-3′; VCAM: forward 5′-GGTATCTGCATCGGGCCTC-3′, reverse 5′-TAAAAGCTTGAGAAGCTGCAAACA-3′; ICAM: forward 5′-AGCTTCGTGTCCTGTATGGC-3′, reverse 5′-TTTTCTGGCCACGTCCAGTT-3′; eNOS: forward 5′-ATCTTCAGCCCCAAACGGAG-3′, reverse 5′-GATCAGACCTGGCAGCAACT-3′; SOD: forward 5′-AGGCATGTTGGAGACTTGGG-3′, reverse 5′-TGCTTTTTCATGGACCACCAG-3′; HO-1: forward 5′-CAACAAAGTGCAAGATTCTG-3′, reverse 5′-TGATTCACATGGCATAAAG-3′; XOD: forward 5′-CTACAGCTTTGAGACTAACTC-3′, reverse 5′-TCTTATGATCTCCTGTTAGGC-3′; p65: forward 5′-TGGGGACTACGACCTGAATG-3′, reverse 5′-GGGGGCACGATTGTCAAAGA-3′; p52: forward 5′-CCGTTGTACAAAGATACGCGG-3′, reverse 5′-CATCCAGACCTGGGTTGTAGC-3′; p50: forward 5′-AATGGGCTACACCGAAGCAA3′, reverse 5′-AGCTCGTCTATTTGCTGCCT-3′; SOD-2: forward 5′-GCTCCCCGCGCTTTCTTA-3′, reverse 5′-GCTGGTGCCGCACACT-3′; GPx1: forward 5′-TATCGAGAATGTGGCGTCCC-3′, reverse 5′-TCTTGGCGTTCTCCTGATGC-3′; catalase: forward 5′-CTCCGGAACAACAGCCTTCT-3′, reverse 5′-ATAGAATGCCCGCACCTGAG-3′; and RPL13A: forward 5′-CACCCTGGAGGAGAAGAGGA-3′, reverse 5′-CCGTAGCCTCATGAGCTGTT-3′. Changes in gene expression were calculated by the 2^−ΔΔCt^ formula using RPL13A as reference gene.

### 2.10. Chemiluminescent Intracellular Xanthine Oxidase Assay

To monitor xanthine oxidase activity, 5 × 10^3^ cells/well were plated in a 96-black well microtiter plate; the day after, cells were incubated at 37°C with CL reaction cocktail solution containing different amounts of cauliflower peptide fractions ranging from 1 × 10^0^-1 × 10^−2^ mg/ml and the CL emission produced after the addition of xanthine (2.0 mM) was monitored for 20 min using the Luminoskan™ Ascent luminometer automatic plate reader (Thermo Fisher Scientific, Roskilde, Denmark). The detailed procedure is reported in a previous paper [25].

### 2.11. Antioxidant Capacity Using a Chemiluminescent (CL) Method

The chemiluminescence method for measurement of antioxidant effect is based on the competition between the reaction of peroxyl radicals with luminol, giving rise to light emission, and the scavenging of peroxyl radicals by antioxidants. Indeed, the addition of a solution of known antioxidants to a glowing steady-state chemiluminescent reaction temporarily quenches light output. The extent of light emission quenching is related to the amount and the strength of antioxidant added. The procedure is reported in [26].

### 2.12. Antimicrobial Activity

The *in vitro* antimicrobial activity of the cauliflower peptide fractions was evaluated towards a panel of reference bacterial strains from the American Type Culture Collection (ATCC) including *Staphylococcus aureus* ATCC 25923, *Staphylococcus epidermidis* ATCC 12228, *Enterococcus faecalis* ATCC 29212, *Escherichia coli* ATCC 25922, *Pseudomonas aeruginosa* ATCC 27853, and *Klebsiella pneumoniae* ATCC 9591 and the yeast *Candida albicans* ATCC 10231.

The peptide fractions were assayed by means of a broth microdilution method as previously described, with minor modifications [27, 28]. Briefly, for antibacterial determinations, a suspension at 0.5 McFarland of each reference strain was diluted 1 : 200 in Mueller-Hinton broth (Sigma-Aldrich) or in Brain heart infusion broth (Biolife) for *E. faecalis* and incubated with tenfold dilutions of cauliflower peptide fractions starting from 1 mg/ml and with gentamicin as reference drug. For antifungal determinations, yeast suspension was diluted 1 : 20 in RPMI-1640 medium (Gibco®, Thermo Fisher Scientific Inc., Waltham, USA), containing glucose 2%, 0.3% levoglutamine, and 0.165 M 3-(N-morpholino)-propanesulfonic acid (MOPS), pH 7.0, and then incubated with tenfold dilutions of peptide fractions starting from 1 mg/ml and with fluconazole, as reference drug. As additional control, cells were incubated in regular medium in the absence of fractions to check both background turbidity and the sterility of the procedure. Following 24 h of incubation at 37°C, microbial growth was determined by adding in each well the WST-8 dye (Microbial Viability Assay kit-WST, Dojindo Laboratories) and measuring the absorbance at 450 nm using the Multiskan Ascent microplate reader (Thermo Fisher Scientific Inc., Waltham, USA). Percentage values of samples at the different experimental conditions were determined as relative to the positive growth controls. Determinations were performed in triplicate and in two independent experiments.

### 2.13. Computational Methods

Molecular docking simulations were performed using the open-source program AutoDock Vina [29] along with AutoDockTools (ADT) [30], a graphical user interface compliment to the AutoDock software suite. In order to run the Vina docking program, both peptidic ligands and protein structure must be first refined and then prepared in a specific file format (.pdbqt). The peptide models of sequences (FKDENGGKLIGF, GNIFDGIQRPL, GYNPSYGARPL, and KWAGGKPEKPILR from fraction 8) were generated using PEP-FOLD3 server that provides a general framework for the structural characterization of peptides and returns in a few minutes the five best models. The models with the lowest energy conformations were selected for docking runs [31–33]. The xanthine oxidase model from bovine milk source (1FIQ) was the most used for docking simulation as reported in literature [34, 35] because of its suitable crystallographic resolution (~2 Å) assuring best docking results. The xanthine oxidase model from bovine milk source (1FIQ) was downloaded from the RCSB protein data bank (http://www.rcsb.org/) and refined by molecular graphic PyMOL software (The PyMOL Molecular Graphics System, Version 2.0 Schrödinger, LLC). Then, ADT was used to create the necessary .pdbqt files of both peptides and xanthine oxidase (XOD) structure that are read by Vina. The identification of candidate regions of the protein surface likely to be involved in the interaction with a peptide sequence required to assist in silico experiments was obtained using PEP-SiteFinder server [36].

### 2.14. Statistical Analysis

Results are expressed as mean ± SD of at least three independent experiments. Differences between the means were determined by one-way ANOVA followed by the Bonferroni multiple comparison test using the GraphPad Prism software, version 6.0 (GraphPad Software Inc., La Jolla, CA), and a *P* value < 0.05 was considered statistically significant.

## 3. Results and Discussion

### 3.1. Peptide Hydrolysate Safety

**Figure 1:**
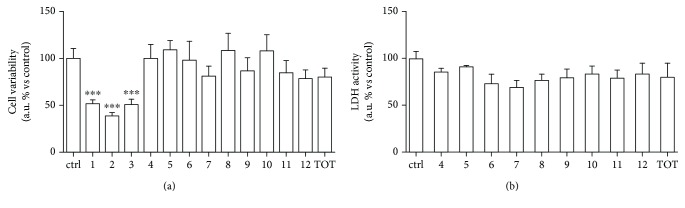
HUVECS were treated with peptide fractions (1-12) and the whole hydrolysate [13], the total (1 mg/ml) for 24 hours. (a) Cell viability was spectrophotometrically detected through formazan production in the presence of dehydrogenases in cells. (b) LDH activity was spectrophotometrically quantified in cellular medium as index of cytotoxicity.

Peptide hydrolysates from cauliflower by-products were obtained by Alcalase®, a low-cost enzyme compatible with large-scale applications [37]. Alcalase® displayed a greater degree of hydrolysis over the other common used enzymes, but it was employed in most cases just to obtain antioxidant and ACE inhibitory peptides [3]. It is certainly interesting to test hydrolysates for less-studied bioactivity since it was suggested that this kind of sample could be a promising source of understudied bioactive peptides [3]; therefore, hydrolysates were subjected to dose-effect safety experiments in HUVECs. Fractions 1, 2, and 3 showed a reduction of cell viability after a 24 h treatment with 1 mg/ml while the others did not affect cell viability. Then, lactate dehydrogenase (LDH), a marker for cell death both *in vitro* and *in vivo*, was quantified in cell culture medium and we did not observe any toxic effect (Figures 1(a) and (b)).

Fractions 4-12 were subsequently investigated to evaluate several unexplored biological activities, such as the protection against endothelial dysfunction and antimicrobial effects.

### 3.2. Peptides from Fractions 8 and 12 Reduce Oxidative Stress through Intracellular Endogenous Antioxidant Enzyme Modulation in HUVEC Cells

**Figure 2:**
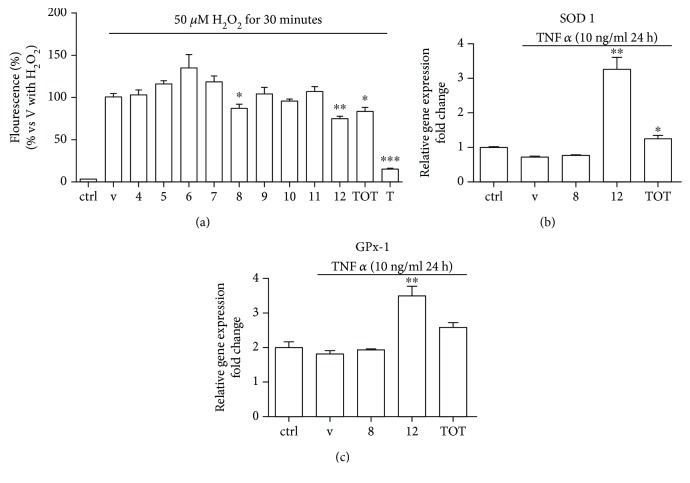
(a) HUVECs were treated with peptide fractions (4-12) and the whole hydrolysate at a concentration of 1 mg/ml for 24 hours and then exposed to oxidative stress generated by 50 *μ*M H_2_O_2_ for 30 min. Treatment with 100 *μ*M Trolox (T) for 24 hours was used as reference. Intracellular ROS levels were measured by means of H_2_DCFDA assay as described in Materials and Methods. HUVECs were pretreated with cauliflower fractions (1 mg/ml) for 8 h before 24 h of exposure to TNF-*α* (10 ng/ml). Total RNA was extracted, and qRT-PCR analysis was performed to determine (b) SOD-1 gene expression and (c) GPx-1 expression. Relative changes in mRNA expression levels were calculated according to the 2^−ΔΔCt^ method using RPL13A as reference gene. Results are expressed as mean ± SEM of three independent experiments. ^∗^ *P* < 0.05, ^∗∗^ *P* < 0.01, and ^∗∗∗^ *P* < 0.001 significantly different from the vehicle (V, DMSO).

Fractions 8 and 12 decreased intracellular ROS levels after acute exposure to H_2_O_2_ (*P* < 0.05 and *P* < 0.01, respectively) (Figure 2(a)), suggesting they exert a protective effect against oxidative stress in the vasculature, process involved in endothelial dysfunction.

Even if H_2_DCFDA is still used to detect intracellular oxidant species from the scientific community, this probe suffers of some limitations as artefactual amplification of the fluorescence intensity via a redox cycling mechanism involving an intermediate radical, DCF^·−^, and responds to changes in intracellular iron signaling or enhanced peroxidase activity [24]. In fact, DCFH does not directly react with superoxide, H_2_O_2_, or nitric oxide. Instead, DCF fluorescence results from oxidation by potent oxidants, such those produced from metal ion- and peroxidase-catalyzed reactions and from decomposition of ONOO^−^. Moreover, DCF-dependent fluorescence can be self-amplified by redox cycling of the one-electron oxidized dye [23]. Indeed, we observed that DCFH probe showed a dose-dependent increase in fluorescence intensity proportional to increase amount of Fe^2+^ (0.3-10 *μ*M) in the presence of 50 *μ*M H_2_O_2_ (Figure S1) in a cell-free system, with a limit of detection (LOD) = 0.6 ± 0.3 *μ*M and a limit of quantification (LOQ) = 2.4 ± 0.3 *μ*M, while we did not observe a direct correlation of DCFH-related fluorescence as a function of increase amount of H_2_O_2_ (0.5-100 *μ*M) (data not shown).

Most peptides derived from hydrolysis of food proteins such as those from milk, egg, meat, wheat, and soy were characterized with chemical assays in cell-free *in vitro* conditions, generally for radical-scavenging activity or for metal chelating activity [38]. However, these methods do not allow evaluating the bioactivity of antioxidant peptides under physiological conditions, in order to establish their real protective roles in diseases. We demonstrated that peptide form cauliflower fractions 8 and 12 reduced intracellular oxidant species, acting as good antioxidants in HUVECs.

To clarify the possible mechanisms of action, we investigated the expression of several prooxidant (NADPH oxidases 2 and 4, lectin-type-oxidized LDL receptor 1, endothelial nitric oxide synthetase, and xanthine oxidase) and antioxidant biomarkers (superoxide dismutases 1 and 2, heme oxygenase 1, catalase, and glutathione peroxidase 1). Peptide fraction 12 at the higher concentration (1 mg/ml) significantly increased superoxide dismutase- (SOD-) 1 and glutathione peroxidase- (GPx-) 1 expression (Figure 2(b)) (*P* < 0.01 and *P* < 0.05, respectively), important intracellular antiatherogenic enzymes that counteract oxidative damage in the vascular endothelium [39, 40].

Among the antioxidant enzymes, SOD-1 is the most abundant and ubiquitous isoform, with a great physiological significance and therapeutic potential in CV diseases because the endothelium is particularly sensitive to oxidant injury [39], so we investigated its expression in HUVECs upon treatment with peptide fractions in the presence of TNF-*α*. Numerous studies suggest that SOD-2 is perhaps one of the most famous NF-*κ*B targets with antioxidant activity in the vascular endothelium [41–44], at least in part via nuclear transcription factor p65 [45]. We investigated SOD-2 gene expression, but we did not observe any significant changes cells upon treatment with peptide fraction 12 in the presence of TNF-*α*.

GPx-1 is the most abundant selenoperoxidase form in mammalian tissues and a key antioxidant enzyme in many cell types including endothelial cells. GPx-1 consumes reduced glutathione to convert H_2_O_2_ to water and lipid peroxides to their respective alcohols [46]; it also acts as an ONOO^−^ reductase [47]. Mice with a disrupted GPx1 gene exhibit increased susceptibility to oxidative stress-inducing agents [48], while induction of this isozyme has been shown to provide protection against oxidative damage in endothelial cells [49] GPx1 deficiency causes endothelial dysfunction [50, 51] and endothelial progenitor cell dysfunction in mice [52]. Furthermore, transgenic GPx1 expression was observed to impair endothelial dysfunction [51].

As some authors observed [53], food-derived peptides can display protective effects by induction of gene expression of proteins that protect cellular components from oxidative stress-induced deterioration; however, to the best of our knowledge, this is the first time that was reported an induction of SOD-1 and GPx-1 expression caused by peptides from cauliflower leaves.

In the endothelium, ROS predominantly arise from the isoforms of NAPDH oxidases 2 and 4 [54]; however, XOD and endothelial nitric oxide synthase (eNOS) play a physiologic role in inflammatory signaling regulation of NO production and vascular function [55]. The oxidative stress generated by these enzymes induces endothelial dysfunction, leading to atherosclerosis, cardiovascular diseases, and metabolic syndrome. Indeed, XOD activity is inversely related to endothelium-dependent vasodilation, since it was located primarily in cells derived from the vasculature and especially in endothelial cells [56]; elevation of XOD activity is associated to poor clinical outcomes [57]. Therefore, to date, XOD is recognized as an important biomarker, incentivizing extensive exploration of inhibition strategies to address disease processes where oxidative stress is contributory, such as cardiovascular disorders [58].

**Figure 3:**
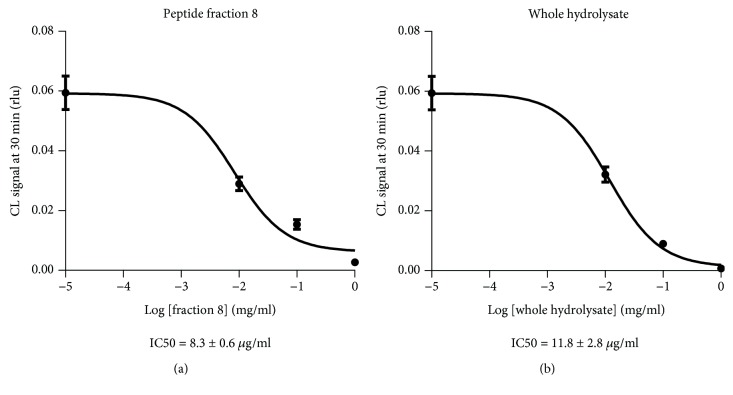
Concentration-response plot of intracellular XOD inhibition obtained by analyzing CL signals after 20 min of incubation with Fe^2+^-EDTA-luminol reaction cocktail in HUVECs treated with peptide (a) fraction 8 and (b) whole hydrolysate (range 1–0.0001 mg/ml).

Peptide fraction treatment did not modulate XOD expression in HUVECs (data not shown) while fraction 8 inhibited intracellular XOD activity (Figures 3(a) and (b)). To quantify intracellular XOD activity, we previously developed an ultrasensitive cell-based biosensor reporting that the xanthine oxidase activity in living endothelial cells (HUVECs) was (6 ± 1) × 10^−7^ mU/ml/cell and the IC50 of oxypurinol, the active metabolite of allopurinol, was 152 ± 76 ng/ml [25]. After 20 minutes of incubation, the intracellular IC50 of fractions 4-12 was evaluated by a dose-response curve, obtaining that fraction 8 has an IC50 = 8.3 ± 0.6 *μ*g/ml (Figures 3(a) and (b)). This cell-based biosensor utilizing whole cells takes into consideration also the bioavailability of the compound, especially the ability to cross cell plasma membranes, so it is more representative and predictive to human situation. Moreover, we previously excluded the possible interferences of all the peptide fractions in the intracellular CL reaction, exploiting an assay based on enhanced chemiluminescent (ECL) detection method, able to reveal different species of ROS [20], demonstrating that fractions 8 and 12 cannot be considered direct ROS scavenger (data not shown). Moreover, to confirm that peptide fractions 8 and 12 act as intracellular antioxidant, we detect intracellular H_2_O_2_ level exploiting cell-based assays with a bioluminescent detection using a boronic probe selective for H_2_O_2_. We treated cells with menadione, one of the simplest quinones, widely used for evaluating the cellular effects of oxidative stress in endothelial cells [59, 60]. The major mechanism caused by menadione is the intracellular production of ROS by redox cycling, where one-electron reduction of O_2_ by the semiquinone form of menadione generates superoxide (O_2_^·−^). O_2_^·−^ is an extremely unstable ROS that rapidly dismutates in the cells to H_2_O_2_ either spontaneously or enzymatically catalyzed by SOD [61]. As it is shown in Figure S2, peptide fractions 8 and 12 reduced menadione-derived intracellular H_2_O_2_. These results confirmed the intracellular antioxidant activities of fractions 8 and 12.

### 3.3. Peptides from Fraction 12 Ameliorate TNF-*α*-Triggered Endothelial Dysfunction in HUVEC Cells

**Figure 4:**
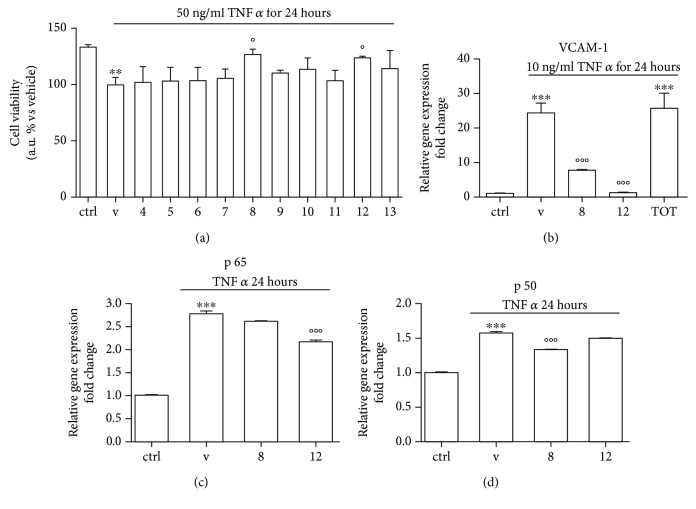
HUVECs were pretreated with peptide fractions (1 mg/ml) for 8 h before 24 h of exposure to TNF-*α* (50 ng/ml). (a) Cell viability was spectrophotometrically detected through formazan production in the presence of dehydrogenases in cells. Total RNA was extracted and qRT-PCR analysis was performed to determine (b) VCAM-1 gene expression, (c) p65 gene expression, and (d) p50 gene expression. Relative changes in mRNA expression levels were calculated according to the 2^−ΔΔCt^ method using RPL13A as reference gene. Results are expressed as mean ± SEM of three independent experiments. ^∗∗^ *P* < 0.01 and ^∗∗∗^ *P* < 0.001 significantly different from the control; °*P* < 0.05, °°*P* < 0.01, and °°°*P* < 0.001 significantly different from the vehicle (V, DMSO).

To better clarify the protective effect of fractions 8 and 12 in respect with TNF-*α*-induced endothelial dysfunction, we evaluated the cell viability in inflammatory conditions. As shown in Figures 4(a) and (b), we observed that TNF-*α* treatment decreased cell viability in HUVECs; the addition of fractions 8 and 12 (1 mg/ml for 24 hours) significantly counteracted the effects induced by TNF-*α* (*P* < 0.05), while lower doses had slight, not significant effect (data not shown). NF-*κ*B signaling is an attractive target for the development of novel anti-inflammatory drugs and the ability of certain small cell-penetrating peptides to enter cells inhibiting NF-*κ*B signaling offer exciting potential also in the clinical setting. Classical NF-*κ*B activity regulates the expression of many genes involved in inflammatory and survival responses, including those encoding cytokines (e.g., IL-1, IL-2, and IL-6), leukocyte adhesion molecules (e.g., E-selectin, ICAM-1, and VCAM-1), and antiapoptotic proteins (e.g., Bcl2, Bcl-XL, and XIAP) [62]. Therefore, the expression of the cellular adhesion proteins VCAM-1 and ICAM-1 was investigated; as it is represented in Figure 4(b), fractions 8 and 12 significantly decreased TNF-*α*-induced VCAM-1 expression (*P* < 0.001) in HUVECs while had no effect in ICAM-1 expression (not shown). To clarify the mechanism of action, the expression of NF-*κ*B-related nuclear transcription factors p65, p50, and p52 was also investigated. As shown in Figures 4(c) and (d), fraction 12 treatment significantly decreased p65 expression while peptide fraction 8 decreased p50 expression (*P* < 0.001 and *P* < 0.01, respectively). p65 protein binds to the promoter of SOD-2 gene [45], so a decrease in p65 expression induced by fraction 12 explains results obtained in SOD-2 expression. NF-*κ*B was the first transcription factor shown to be redox-regulated [63] and it has been demonstrated that overexpression of SOD-1 suppressed ischemia-induced activation of NF-*κ*B through a decrease in nuclear translocation protein levels [64]. It has been also shown that transfection of endothelial cells with SOD-1, but not catalase, inhibited NF-*κ*B signaling and expression of VCAM-1 induced by TNF-*α* [65].

NF-*κ*B activation is regulated by reactive species [63], and ROS derived from intracellular XOD activity is implicated in heart failure [66] possibly through NF-*κ*B-related p50 modulation.

Therefore, a decrease in intracellular oxidative stress by fraction 8 and 12 treatment can inhibit NF-*κ*B signaling in endothelial cells, which can suppress its downstream effects. This notion was supported by the effects of fractions 8 and 12 on VCAM-1 mRNA level measured by qPCR.

Notably, the anti-inflammatory activity of a peptide derived from ovotransferrin, which is present in the albumen of eggs, was recently explained through the NF-*κ*B-related p50 and p65 inhibitions [67].

Increased reactive oxygen species (ROS) production together with increased adhesion molecules and thrombogenic tissue factor expression on endothelial cells has a key role in proatherogenic mechanisms [68]. Therefore, peptides able to decrease the expression of inflammatory biomarkers could be useful for reducing the severity of atherosclerosis progression.

### 3.4. Peptides from All of the Fractions Do Not Show Any Antimicrobial Activity

The characterization of several plant protein hydrolysates demonstrated that they have antimicrobial activity, thus qualifying as functional foods [69], but we did not observe inhibitory activity of the cauliflower peptide fractions towards the human pathogenic bacteria and yeast strains selected in the present study.

### 3.5. Identification of Bioactive Peptides from Fractions 8 and 12

Fractions 8 and 12, the most active ones, were analyzed by nano-HPLC-MS/MS method. The obtained MS/MS raw files were searched by the Proteome Discoverer software to obtain peptide sequences. The identified peptides were manually validated taking into consideration only the most abundant peptides in both fractions; peptide abundance is related to their area; thus, peptides were filtered according to it and only the ones with an area larger than 10^7^ and higher score were accepted. A total of 181 peptides were identified after this manual validation. The complete list of identified peptides coming from the two most active fractions from the first chromatographic dimension, with sequence and related data, is reported in Supplementary Material S2.

After that, an in silico analysis using PeptideRanker [70] was carried out to further mine data. In this way, each peptide was assigned a score based on the probability of being bioactive, probability that the built-in N-to-1 neural network computed on the basis of the peptide primary sequence (the complete list of probability scores assigned to each identified peptide is reported in Supplementary Material S2). Such algorithm is capable of predicting the bioactivity of peptides because of the general features that different bioactive peptide functional classes have in common; therefore, PeptideRanker represented a useful tool to select the most probable bioactive peptides. Since the software labelled as “bioactive” any peptide possessing a score above the 0.5 threshold, we applied a higher 0.7 threshold in order to reduce the number of false positive hits. After such filtering of peptide scores, most peptide sequences were rejected. Twenty-three peptides have shown a score higher than 0.7 in fraction 12, and among these, only one has shown a probability higher than 0.9, namely, IDNIFRF. As it is possible to see in Supplementary Material S2, only four potentially bioactive peptides come from fraction 8, and all the others belong to fraction 12. The treatment with fraction 12 increased SOD-1 and reduced VCAM-1 expression in the presence of TNF-*α*. Both genes are modulated by NF-*κ*B signaling [43, 71], suggesting that one or more peptides in fraction 12 could act as inhibitor of NF-*κ*B pathway machinery. Several bioactive peptides have been described in literature as inhibitors of classical NF-*κ*B signaling by either disrupting the IKK complex or by inhibiting critical events downstream of IKK*β* [72]. In addition, several peptides that target upstream intermediates in the NF-*κ*B pathway or other signaling mechanisms, such as the ERK and JNK pathways, have also been developed. IDNIFRF seems the most promising candidate due to the presence of both the cationic (arginine) and hydrophobic side chains (isoleucine) that can facilitate its uptake across the plasma membrane. An in silico determination of the hydrophobic character of this peptide was performed exploiting Peptide 2.0 ProtParam tool of ExPASy bioinformatics resource portal and peptide synthesis and proteotypic peptide analyzing tool of Thermo Scientific confirmed a hydrophobicity of 30.90, with an aliphatic index of 111.43 and a grand average of hydrophobicity (GRAVY) of 0.443 [73]. These results suggested that IDNIFR is an adequate hydrophobic peptide able to cross plasma membranes. However, to the best of our knowledge, more studies are needed to determine its efficacy as NF-*κ*B signaling modulator.

### 3.6. Molecular Docking Simulations for Xanthine Oxidase

**Figure 5:**
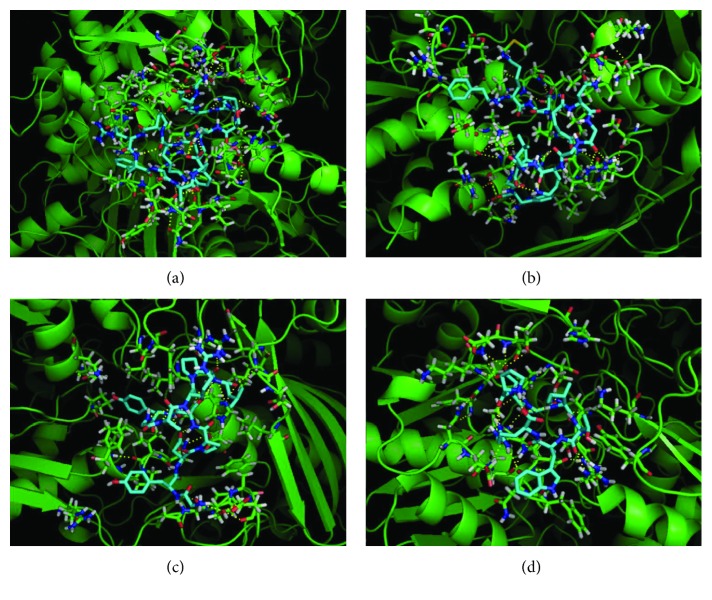
Picture of peptides docked in XOD enzyme (1FIQ), showing polar interactions within active sites for (a) GDSNPSNPKPRFGAY, (b) FKDENGGKLIGF, (c) GYNPSYGARPL, and (d) PDSITWR sequences, calculated by AutoDock Vina and elaborated by PyMOL software.

**Figure 6:**
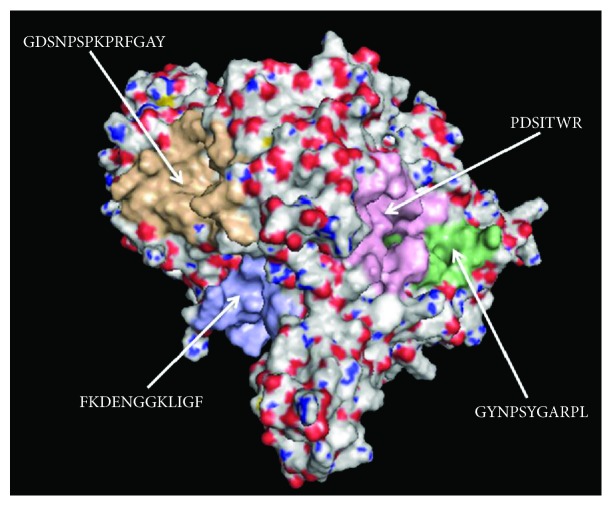
Picture of XOD enzyme polar surface, showing that each sequence (GDSNPSNPKPRFGAY, PDSITWR, FKDENGGKLIGF, and GYNPSYGARPL) interacts with a different site of XOD, calculated by PEP-SiteFinder server and elaborated by PyMOL software.

To validate the experimental work conducted to determine the intracellular XOD inhibition, molecular docking simulations have been used as a tool to augment the molecular level interpretation of the data. In particular, to seek the molecular explanation of the inhibition behavior of fraction 8 against XOD activity in the cell-based assay, we undertook a series of molecular docking studies using AutoDock Vina. The in silico calculations provided nine best output results for each docking run. From the analysis of docking study, the peptide sequence GDSNPSNPKPRFGAY showed the best docked conformation (Figure 5) with the lowest energy affinity of -28.03 kJ/mol than other peptides (Table 1). The docking results indicated that medium-weak interactions between protein surface and peptides exist. Then, theoretical dissociation constants *K*_i_ (ranged from 20 to 52 *μ*g ml^−1^) for peptide sequences were calculated from docking outputs and compared with experimental *K*_i_, derived from cell-based assay results. The *in silico* simulations referred only to a single isolated peptide and did not consider cell permeability of inhibitors. Moreover, the discrepancy between experimental and computational *K*_i_ values can be explained considering a positive synergic effect of the four inhibitors [74]. Indeed, Figure 5(b) shows four different regions of the protein surface involved in the interaction with each peptide, respectively. The synergistically interaction of the four sequences to different sites of XOD can justify the increased inhibitory effect, as observed in *in vitro* assays. Molecular docking simulations demonstrated that the trend of in silico prediction of peptide effects is in agreement with experimental *in vitro* results and confirmed inhibitory effect of cauliflower fraction 8 on XOD activity as obtained from cell-based assay, providing a theoretical explanation of molecular inhibition mechanism by the identification of protein-peptide interactions (Figures 5 and 6).

## 4. Conclusions

We developed an innovative combined (bio)analytical approach, based on a rationale recovery of bioactive peptides from cauliflower waste through an advance peptidomic-based strategy integrated with an *in vitro* activity characterization utilizing highly predictive cell-based bioassays. Our study suggests that cauliflower peptides from two fractions possess antioxidant and anti-inflammatory effects in the vasculature, at least in part through inhibition of intracellular XOD activity and modulation of SOD-1 and VCAM-1 expression. *In silico* analysis showed that four peptides from fraction 8 and one from fraction 12 could be the most probable bioactive candidates exerting protective effects against endothelial dysfunction. Moreover, one peptide from fraction 8 able to synergistically inhibit XOD was detected through a detail *in silico* docking analysis.

Advancements in the biopharmaceutical industry have resulted in the development of several new peptide-based therapeutics; to the best of our knowledge, this is the first attempt to determine biological effects of peptides from cauliflower waste, in order to evaluate their possible application into valuable functional components in nutraceutics and pharmaceutics as well as in animal feed.

Oral administration is most preferred because of patient compliance and acceptability; however, to exercise their effects in the target organ, peptides need to remain intact during the digestive process. To solve this crucial issue, several approaches including chemical modifications (lipidation), physical methods (microencapsulation), use of mucoadhesive polymers, formulation design, and use of enzyme inhibitors have been developed to improve their bioavailability after oral ingestion [58].

This translational (bio)analytical approach represents a smart and powerful tool that allows to open new perspectives on the possibility to find new uses of waste, even outside the agricultural field, contributing to the creation of sustainable value chains in the farming and processing sectors.

## Figures and Tables

**Table 1:** Comparison between inhibition constant values (*K*_i_) derived from cell-based assay and in silico molecular docking simulations of bioactive peptides in cauliflower fraction 8.

*K*_i_ calculated from cell-based assay	Peptide sequence	Energy affinity	*K*_i_ calculated from in silico simulation results
8.6 *μ*g ml^−1^	GDSNPSNPKPRFGAY	-28.03 kJ/mol	20 *μ*g ml^−1^
	PDSITWR	-25.10 kJ/mol	34 *μ*g ml^−1^
	GYNPSYGARPL	-25.52 kJ/mol	40 *μ*g ml^−1^
	FKDENGGKLIGF	-25.10 kJ/mol	52 *μ*g ml^−1^

## Data Availability

The in vitro data obtained in human umbilical vein endothelial cells used to support the findings of this study are included within the article. The description of the in vitro method to determine antimicrobial activity is included within the article; however, no data are available since peptide fractions did not show any significant antimicrobial activity. Previously reported data regarding the multidimensional liquid chromatography characterization of peptide fractions were used to support this study and are available at DOI: 10.1016/j.jff.2018.02.022. These prior studies are cited at relevant places within the text as references [17]. The data obtained by peptidomic analysis and bioinformatics used to support the findings of this study are included within the article and in supplementary information files 1 and 2.

## Supplementary Materials

Supplementary 1 Supplementary Material S1: a detail description of the entire peptidomic workflow, procedures, and results regarding the intracellular oxidants measurement were reported.

Supplementary 2 Supplementary Material S2: the complete list of identified peptides coming from the two most active fractions from the first chromatographic dimension, with sequence and related data, was reported.
