# Supplementary material for: Peptides from Cauliflower By-Products, Obtained by an Efficient, Ecosustainable, and Semi-Industrial Method, Exert Protective Effects on Endothelial Function
Source: Oxid Med Cell Longev. 2019 Feb 6;2019:1046504. doi: 10.1155/2019/1046504 (PMC6381550; doi:10.1155/2019/1046504)
Supplement: Supplementary 1 — Supplementary Material S1: a detail description of the entire peptidomic workflow, procedures, and results regarding the intracellular oxidants measurement were reported. [file 1046504.f1.pdf]

SUPPLEMENTARY MATERIAL S1

1.1 Chemicals and standards

Alcalase® enzyme from Bacillus licheniformis, Tris (hydroxymethyl) aminomethane (Tris-HCl), trifluoroacetic acid (TFA) and all other chemicals and solvents, of the highest grade available, were purchased from Sigma Aldrich (St. Louis, MO, USA), unless otherwise stated. Pierce BCA Protein Assay Kit was bought from Thermo Fisher Scientific. Ultrapure water (resistivity 18.2 MV cm) was obtained by an Arium 611 VF system from Sartorius (Göttingen, Germany). Solid phase extraction (SPE) C18 cartridges were Bond Elut (Varian, Palo Alto, CA, USA).

1.2 Plant material, protein extraction and precipitation

The cauliflower (*Brassica oleracea L. var. botrytis*) was provided by local farmers. Cauliflower by-products consisting of leaves and stem have been isolated, chopped with a sharp stainless steel knife into small pieces, lyophilized and finally ground to a fine powder. Protein extraction was simplified with respect to from our first work [1]. The protocol was carried out starting from 1 kg of lyophilized cauliflower by-products. Briefly, samples were extracted with 5 L of a buffer consisting of 50 mmol L^{-1} Tris-HCl (pH 8.8), 15 mmol L^{-1} KCl, 20 mmol L^{-1}. Briefly, the samples were incubated on ice for 1 h with intermittent shaking (1 min every 15 min), followed by 1 h of sonication; the two steps were repeated twice. Finally, the insoluble material was removed by centrifugation for 15 min at 4,000×g. The supernatants containing proteins were transferred into new bottles and subjected to protein precipitation at pH 3.5 using 1.0 M HCl. The obtained pellets were collected by centrifugation (4,000×g for 15 min) and dissolved in 100 mL of water pH 8.8. Proteins samples were quantified by the BCA assay, using bovine serum albumin (BSA) as standard and stored at −80 °C until digestion.

1.3 Protein digestion

After quantification, the cauliflower by-product protein pellet was hydrolysed by Alcalase®. A total of 10 g of proteins were digested with Alcalase® (1:10, enzyme: protein ratio) and the samples were incubated at 60° C for 4 h. Enzymatic hydrolysis was quenched by decreasing the pH to 2 with TFA. The resulting peptide mixture was stored at -20 °C until analysis.

1.4 Purification of the cauliflower waste hydrolysate by RP-HPLC

The aqueous protein hydrolysate was fractionated using solid phase extraction (SPE) followed by semi-preparative reverse phase-high performance liquid chromatography (SP-RP-HPLC) as previously described [1]. A 1% (w/v) protein hydrolysate (in HPLC-grade H_2O) solution was applied to a pre-activated SPE column (Bond Elut 1 g/6 ml C18, Varian, Palo Alto, CA, USA). Peptide fractions were eluted from the cartridge using 15 ml HPLC grade H_2O and 70% (v/v) ACN. The eluate was dried using a solvent evaporator (Genevac, EZ-2 Plus, Genevac Ltd., Ipswich, UK).

Hydrolysed peptides were fractionated using an Xbridge BEH preparative C18 5µm OBD 19 x 250 mm (Waters) connected to Shimadzu Prominence LC-20A system, including a CBM-20A controller, two LC-20 AP preparative pumps, a DGU-20A3R online degasser. A SPD-20A UV with a preparative cell (0.5 mm) was used as detector. As autocollector a FRC-10A Shimadzu was employed. Data acquisition was performed by the LabSolution version 5.53 software (Shimadzu, Kyoto, Japan). The detector was set at 214 nm.

The sample was eluted with a flow rate of 17 mL min^{-1} using ddH_2O/TFA (99.9/0.1, *v*/*v*) as mobile phase A and MeOH/TFA (99.9/0.1, *v*/*v*) as mobile phase B. The gradient started from 25% B, was kept for 1 min and then increased to 50% in 19 min; finally, B was increased to 95% and maintained constant for 5 min. The column was re-equilibrated for 6 min. Twelve fractions were collected every 2 minutes (except for fraction 1 and 12, as it possible to see in Figure 1). Collected peptide fractions (F1-12) were subjected to bioactivity tests to identify the most active ones.

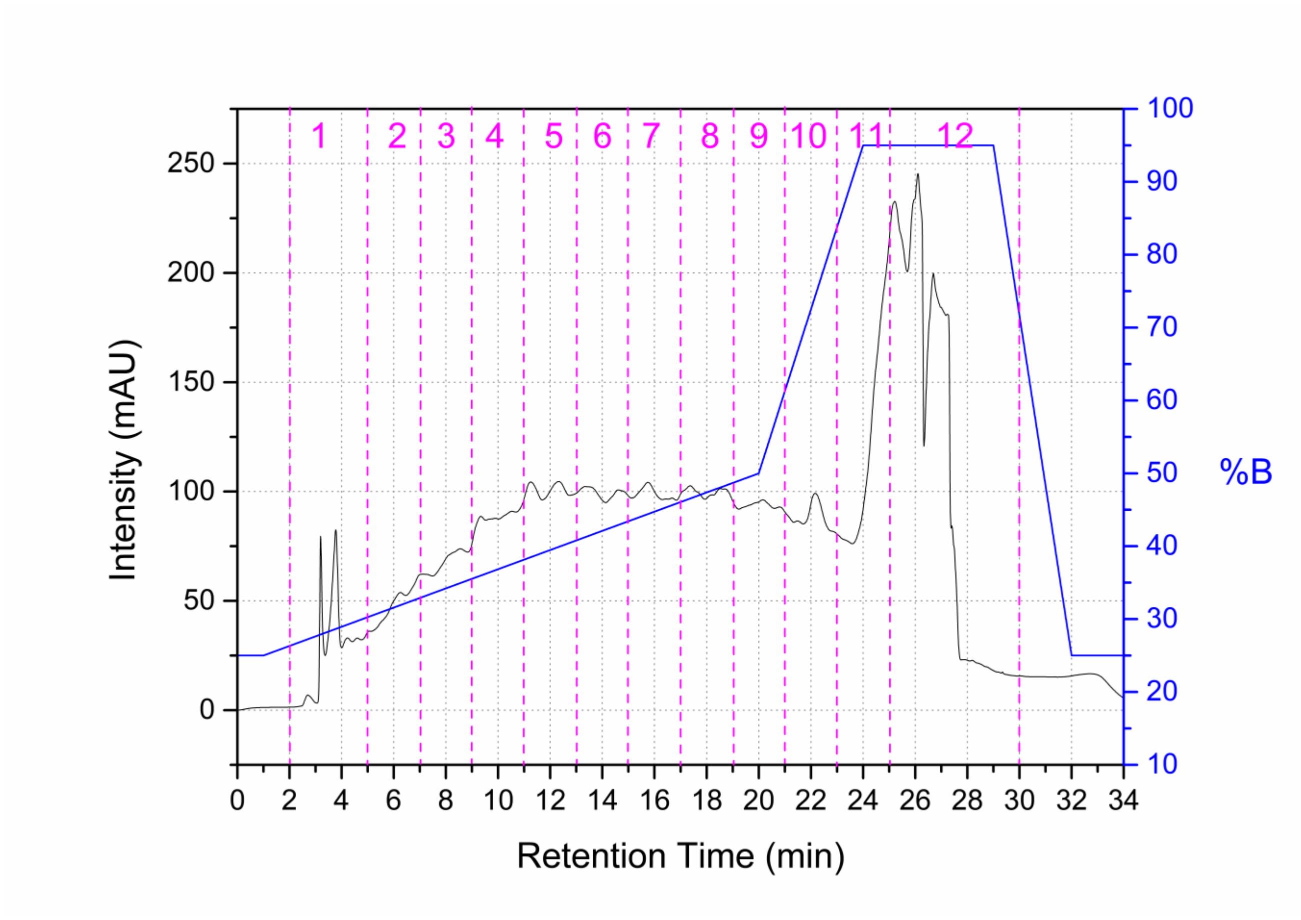

Figure 1. Fractionation scheme adopted for preparative RP-HPLC of cauliflower waste hydrolysates.

1.5 NanoHPLC-MS/MS Analysis

The most active fractions, namely F8 and F12, were analysed by nanoHPLC coupled to MS/MS. The analysis was performed on a Dionex Ultimate 3000 (Dionex Corporation Sunnyvale, CA, U.S.A.) directly connected to a LTQ-Orbitrap XL mass spectrometer (Thermo Scientific, Bremen, Germany) by a nanoelectrospray ion source. Peptide mixtures were on-line enriched on a 300 µm ID × 5 mm Acclaim PepMap 100 C18 (5 µm particle size, 100 Å pore size) precolumn (Dionex Corporation Sunnyvale, CA, U.S.A.), employing H_2O/ACN, 98/2 (*v*/*v*) with 0.1% (*v*/*v*) TFA at a flow-rate of 10 µL min^{-1}. Then, peptide mixtures were separated by RP chromatography using a 25 cm long fused silica nanocolumn (75 µm ID, in-house packed with Acclaim-C18 2.2 µm microparticles). The LC gradient used H_2O/HCOOH (99.9/0.1, *v*/*v*) as phase A and ACN/HCOOH (99.9/0.1, *v*/*v*) as phase B.

The gradient comprised 5 min isocratic step with phase B at 2%, then phase B was linearly increased to 7% in 10 min, to 15% in 85 min, to 21% in 45 min, to 30% in 30 min and to 50% in 10 min. The column was washed for 20 min at 80% phase B, and equilibrated for 45 min. The LTQ-Orbitrap XL hybrid mass spectrometer was operated in positive ionization mode in the *m/z* range 350–1,800, in top 5 data dependent mode and collision inducted dissociation. Spectra acquisition was performed as previously described [1].

1.6 Database search and peptide identification

All raw files from Xcalibur software (version 2.2 SP1.48, Thermo Fisher Scientific) were submitted to Proteome Discoverer software (version 1.3, Thermo Scientific) with the Mascot search engine (v.2.3, Matrix Science) for peptide/protein identification. The searches were performed against the proteome of *Brassica olearacea* species downloaded from Uniprot (http://www.uniprot.org/) on February 2016 (60280 sequences with 20896061 residues). No enzyme was chosen and the minimum required peptide length was set to 5 amino acids. No modification was chosen. The potential bioactive peptides were subsequently filtered using three parameters: a Mascot-score larger than 30, an area of the integrated peak larger than 5E7 and the score of PeptideRanker [2] larger than 0.7. The potential peptide candidates were searched against the BIOPEP database (http://www.uwm.edu.pl/biochemia/index.php/en/biopep) and PepBank (http://pepbank.mgh.harvard.edu/) to determine their novelty.

1.7 DCFH fluorescent detection to evaluate H_2O_2 and Fe^{2+} level in a cell-free system

Briefly, H_2O_2 (0,1-100 μM) and Fe^{2+} (0,1-10 μM) solutions were prepared in PBS. 100 μl of each solution was dispensed in a black 96 wells (Fe^{2+} solutions in presence of 50μM H_2O_2). Finally, DCFH was injected to obtain a final concentration of 5 μM, and fluorescence was measured for 30 minutes using a microtiter plate reader (Varioskan™ Flash Multimode Reader, Thermo Fisher Scientific).

Excitation wavelength was 485 nm and emission wavelength 535 nm.

Figure S1

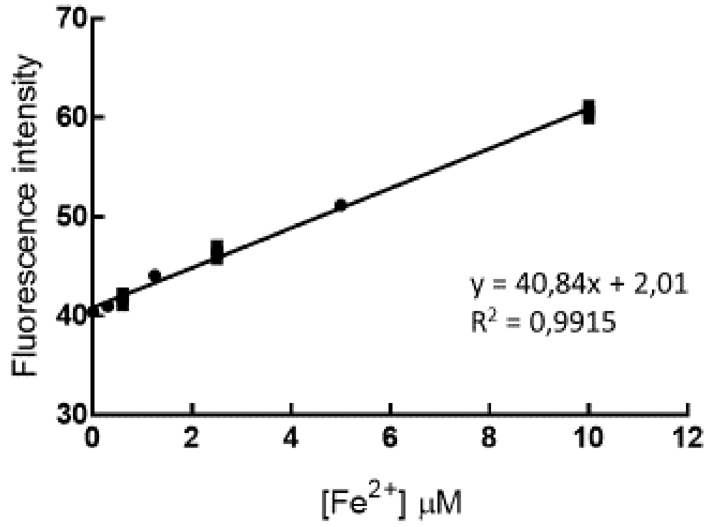

Figure S1. Cell-free system. Calibration curve obtained by analysing fluorescence intensity after 30 min of Fe^{2+} (0,1 -10 μM) addition. The plot was linear up to 10 μM, with a limit of detection (LOD) and a limit of quantification (LOQ) of 0.6 ± 0.3 μM and 2.4 ± 0.3 μM, respectively.

1.8 Intracellular H_2O_2 bioluminescent detection

ROS-Glo™ H_2O_2 assay kit by Promega (Madison, WI, USA) were used to test intracellular H_2O_2 levels in HUVECs (10×10^3 cells/well) in the presence of menadione (Sigma Aldrich, St Louis, MO, USA) (10-100 μM) after cell treatment with peptide fractions for 24 hours, according to the manufacturer's instruction. Then, luminescence was determined every 5 min for a total of 60 min using the Luminoskan™ Ascent luminometer automatic plate reader (Thermo Fisher Scientific, Roskilde, Denmark).

Figure S2

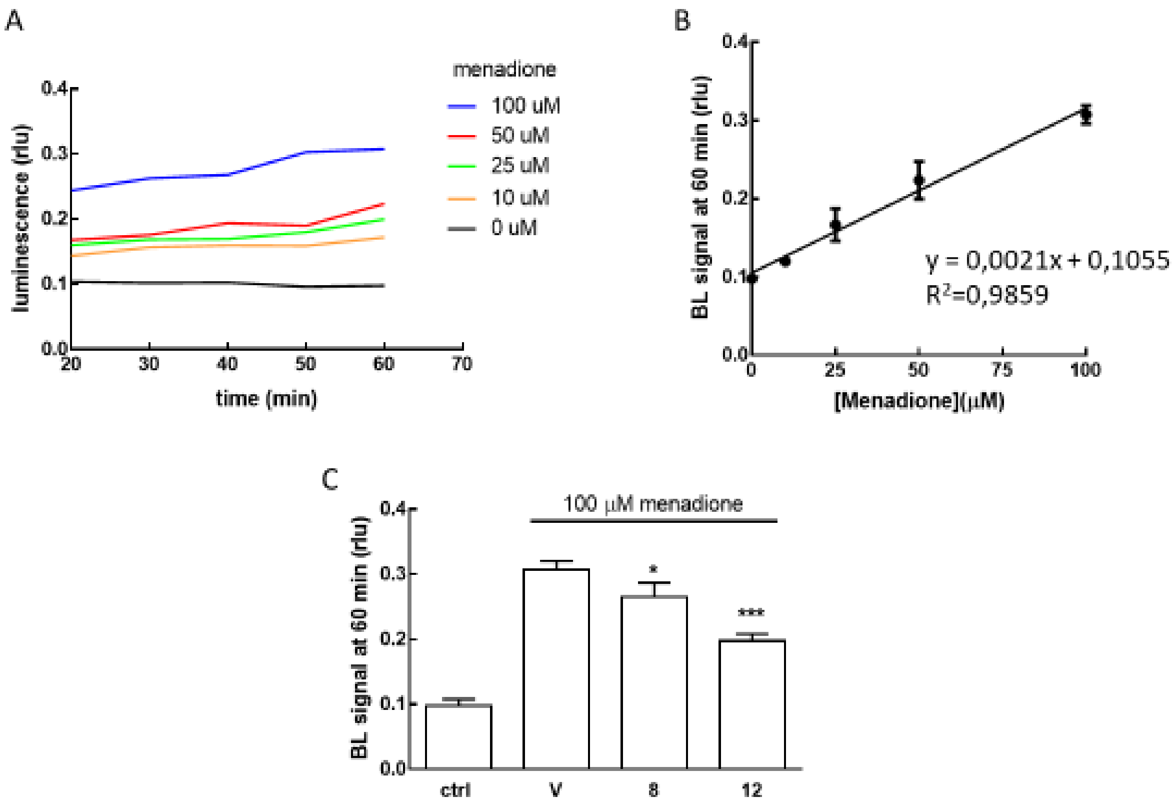

Figure S2. Cell-based assays. (A) BL kinetic profiles of the light emission (HUVECs, 10 × 10^3 cells/well) obtained from the analysis of different concentrations of menadione (10-100 µM) at 37 °C for 60 min. (B) Concentration-response plot of intracellular H_2O_2 obtained by analyzing BL signals after 60 min of incubation with menadione (10-100 µM) in HUVECs. (C) Intracellular H_2O_2 levels were measured by means of bioluminescent cell-based assay as previously described, in HUVECs treated with peptide fraction 8 and 12 in the presence of menadione 100 µM for 60 min.

References

(1) Zenezini Chiozzi, R.; Capriotti, A. L.; Cavaliere, C.; La Barbera, G.; Piovesana, S.; Laganà, A. Identification of three novel angiotensin-converting enzyme inhibitory peptides derived from cauliflower by-products by multidimensional liquid chromatography and bioinformatics. *J. Funct. Foods* **2016**, *27*, 262–273.

(2) Mooney, C.; Haslam, N. J.; Pollastri, G.; Shields, D. C. Towards the Improved Discovery and Design of Functional Peptides: Common Features of Diverse Classes Permit Generalized Prediction of Bioactivity. *PLoS One* **2012**, *7* (10), e45012.
